# Epidermoid cyst in ureter: A case report

**DOI:** 10.1097/MD.0000000000030254

**Published:** 2022-09-16

**Authors:** Qiang Jing, Xin Wang, Xiaobin Yuan, Fan Liu, Xuhui Zhang

**Affiliations:** a Department of Urology, First Hospital of Shanxi Medical University, Taiyuan, Shanxi, China.

**Keywords:** case report, sparing urinary tract, ureterocyst epidermoid

## Abstract

Uro-originated epidermoid cysts, especially located in the upper urinary tract are rare and only 4 cases of epidermoid cyst occurring in the upper urinary tract have been reported worldwide so far. In the previous cases, the cysts were diagnosed as tumors and organs were removed.

**Patient concerns and diagnosis:**

We report a case of epidermoid cyst in ureter in a 48-year-old female patient admitted to the local hospital suffering from paroxysmal pain in the right hypochondriac for 8 years.

**Interventions and outcomes:**

She underwent right ureteroscopy in order to rule out the possibility of urinary epithelial carcinoma. The tumor was pathological diagnosed as a benign ureterocyst epidermoid. Postoperatively, the patient showed good recovery. During the 24-month follow-up period, the patient remained well and free of complications.

**Conclusion:**

This case illustrates that benign epidermoid cysts can appear in the ureter, although it is extremely rare. It also indicates that perioperative examinations must be exhaustive to avoid the further injury to the patients.

## 1. Introduction

An epidermoid cyst is a benign slow-growing epithelium tumor originated from the dermis or subcutaneous. The most common locations are the head and body cadres, limbs, retroperitoneal and pelvic cavities.^[1]^ Epidermoid cysts of genitourinary system are rare, with occurrences in the testis being the most reported, but they are extremely rare in the upper urinary tract such as in the kidney and ureter.^[2]^ Here we present a unique case of an epidermoid cyst in the middle section of the ureter, while providing ideas for the diagnosis and treatment of ureteral epidermoid cysts.

## 2. Case presentation

**Figure 1.**
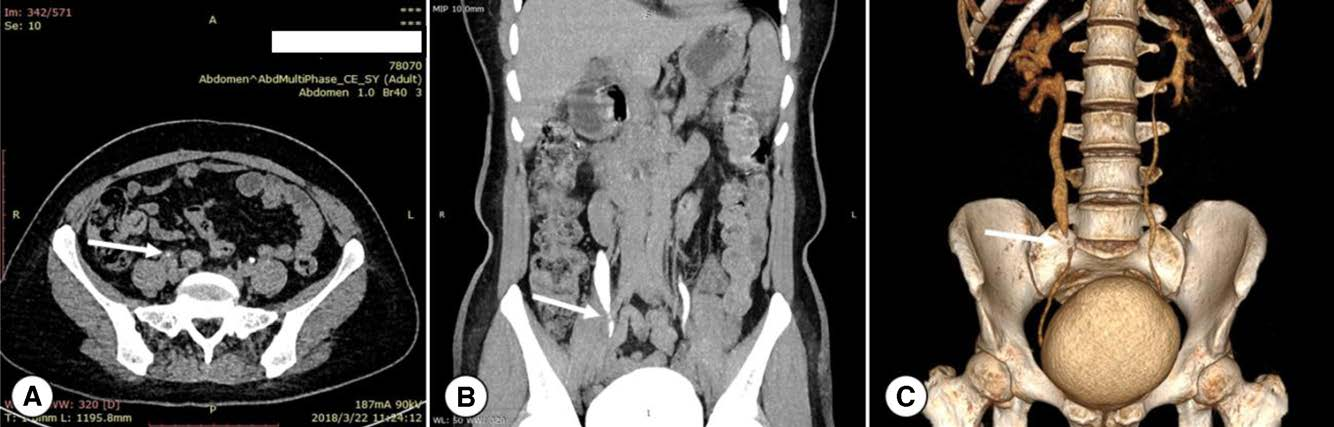
A. Computerized tomography (excretory phase) demonstrated filling defect of the diseased ureter (white arrow). B. Mip (Maximum Intensity Projection) revealed the ureterostenosis, marked by the white Arrow. C. Reconstructed digital model showed ureterectasia and hydronephrosis in supraureteral obstruction.

**Figure 2.**
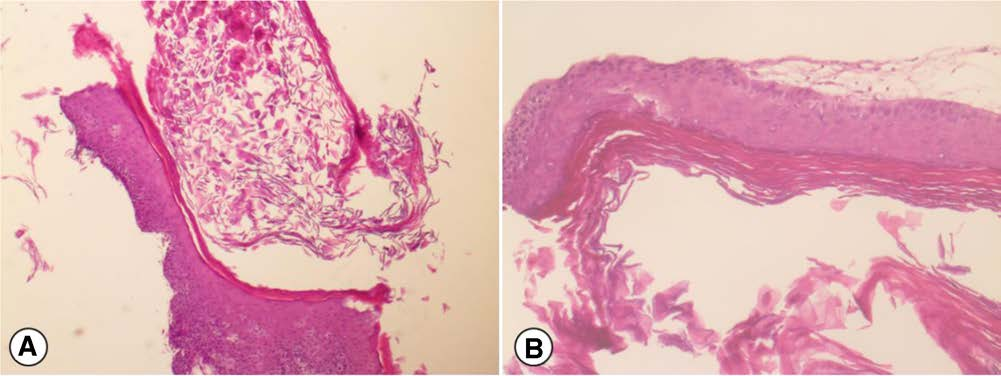
Hematoxylin-eosin staining. A. Low power view (×40) showing cyst wall. B. High power view (×100) showed the cystic wall lined by stratified squamous epithelium, a granular layer containing keratin and subcutaneous fibrous tissue hyperplasia without any nuclear atypia.

A 48-year-old woman was admitted to our hospital with paroxysmal pain in the right hypochondriac for 8 years without other concomitant symptoms. She had a history of diabetes and hypertension. During the incubation period, she was suspected to suffer from ureteral calculus and underwent extracorporeal shock wave (ESWL) more than once at the local hospital. The patient’s laboratory examination, such as renal biochemical parameters and urine analysis, were normal. Plain and contrast-enhanced computerized tomography (CT) of urinary system indicated a stenotic change in the middle segment of the right ureter (at about S1 level), and a tumor was detected in that site. The ureter above the obstructed site was dilated concurrently with the ureter wall significantly thickened (Fig. 1). SPECT renal dynamic imaging revealed there was mild renal insufficiency, and the left glomerular filtration rate (GFR) in 28.5 ml/min, other 33.8 ml/min. There was no abnormal findings in the outcomes of voided urine cytology and fluorescence in situ hybridization after testing 3 times. Right ureteroscopy was performed in order to rule out the possibility of urinary epithelial carcinoma. A cyst was seen in the middle part of the ureter under ureteroscope, we then excised it by using a holmium laser (energy 0.8 J, frequency 25 Hz). After the excision, an F6 double J tube was indwelled to prevent the ureterostenosis. The final histopathological examination showed cystic wall lined with stratified squamous epithelium, a granular layer containing keratin and subcutaneous fibrous tissue hyperplasia without any nuclear atypia (Fig. 2). The patient provided informed consent for the publication of her clinical data and accompanying images. Postoperatively, the patient showed good recovery. During the 24-month follow-up period, the patient remained well and free of complications. The patient received the treatment that is currently routine and hence no ethical approval was required.

## 3. Discussion

Epidermoid cyst is a kind of benign tumor composed of epithelium, intact wall and obvious granular layer. Under the microscope, the cystic structure consists of keratin epithelial cells, scales, and cholesterol crystals. The etiology of epidermoid cysts has not yet been explored in depth,^[3]^ some scholars think that an epidermoid cyst is formed when embryonic neural tube is closed with the ectoderm, gradually growing into tumor, namely the skin epidermal layer residues in the site occurring as a cyst. With the renewing and shedding of keratinized cells, the contents of cyst gradually increased and eventually lead to the tumor formation. Some experts also believe that epidermal cells are implanted in tissues or organs during trauma and grow into epidermoid cysts. Epidermoid cysts can occur at any age, more frequently in the head^[4]^ and body cadres than in the extremities, retroperitoneal and pelvic cavities. They can also present in the skull, finger bones, spleen, breasts and other parts of the body.^[5]^ Epidermoid cyst of the genitourinary system is rare. There are many cysts occurring in testicles,^[6]^ but those occurring in the upper urinary tract, such as the kidney and ureter, are extremely rare. Only 4 cases of epidermoid cyst occurring in the upper urinary tract have been reported worldwide to date (Table 1).^[2,3,7,8]^

**Table 1:** Summarizing of epidermoid cyst in ureter.

	No.	Age/gender	Size	Location	CT	Diag.	Treatment
Boeminghaus F, et al (1971)	Case 1	28/female	–	Renal pelvis	–	Renal pelvis tumor	Nephroureterectomy
Salgarello G, et al (1980)	Case 2	3/female	3 cm	Renal pelvis	–	Undifferentiated	Enucleation
Gokee G, et al (2003)	Case 3	55/female	9 cm	Renal calix	Solid mass	Transitional cell carcinoma	Nephroureterectomy
Hironori Ishizaki, et al (2007)	Case 4	72/male	2 cm	Ureter	calcified mass with partial enhancement	Ureteral stone	Nephroureterectomy

CT = computerized tomography.

Epidermoid cysts located in the upper urinary tract, especially in the ureter, often cause an obstructive effect, resulting in hydronephrosis and symptoms such as renal colic and hematuria. In this case, we reported that the patient suffered from paroxysmal pain in the right lumbar back several times in the last 8 years. The patient was misdiagnosed as a having a right ureteral calculi in the local hospital, and underwent extracorporeal shock wave lithotripsy.

Currently, the medical imaging examinations have no adequate specificity and sensitivity to diagnose an epidermoid cyst in ureter accurately. The occurrence of epidermoid cysts in the previous reports were misdiagnosed as renal tumors or urothelial tumors, and these patients underwent nephrectomy or nephroureterectomy.

Grayscale ultrasonography shows a well-circumscribed hypoechoic nodule,^[9]^hypodense, nonenhanced lesions on CT scans,similar to fat-tissue density. Magnetic resonance imaging (MRI) shows inhomogeneous hypointense T1 signal and hyperintense T2 signal. These cysts are often nonenhanced but may show minimal peripheral contrast enhancement.^[10]^ Given the narrow ureteral cavity and the nature of small lesions, CT diagnosis effect is inferior to MRI. In this present case, we finally decided to perform the ureteroscopy plus biopsy to clarify the nature of the pathological change for the absence of cytological evidence preoperatively.

Although epidermoid cyst is a benign disease, there is a 2% probability of progression to squamous cell carcinoma and 3% chance of recurrence after surgery.^[11]^There are many treatment available, including conservative observation, simple cystectomy, and radical resection. In this case, the patient suffered from the chronic urinary tract obstruction that eventually resulted in a slight impairment of the right renal function. In addition, the patient had a history of high blood pressure for many years and the left renal function was also mildly damaged, therefore relieving the obstruction, reserving the renal function and regular follow-ups might be more beneficial for patients with long-term survival.

## 4. Conclusion

Ureteral epidermoid cysts are extremely rare and easy to be misdiagnosed preoperatively, leading to improper treatment. Currently, the medical imaging examinations have no adequate specificity and sensitivity to diagnose this disease accurately. However, ultrasonography shows a well-circumscribed hypoechoic nodule, and seems to improve diagnostic performance. Moreover, ureteroscopy is feasible when necessary.

## Acknowledgments

We thank Dr Zhang and Dr Yuan for performing the surgery. We would like to thank Editage (www.editage.com) for English language editing.

## Author contributions

Writing—original draft: Qiang Jing

Writing—review & editing: Xin Wang and Fan Liu
